# Defects in oxide surfaces studied by atomic force and scanning tunneling microscopy

**DOI:** 10.3762/bjnano.2.1

**Published:** 2011-01-03

**Authors:** Thomas König, Georg H Simon, Lars Heinke, Leonid Lichtenstein, Markus Heyde

**Affiliations:** 1 Fritz-Haber-Institut der Max-Planck-Gesellschaft, Faradayweg 4-6, 14195 Berlin, Germany

**Keywords:** aluminum oxide, charge state, contact potential, defects, domain boundaries, dynamic force microscopy, frequency modulation atomic force microscopy, Kelvin probe force microscopy, magnesium oxide, non-contact atomic force microscopy, scanning tunneling microscopy, thin ﬁlms, work function

## Abstract

Surfaces of thin oxide ﬁlms were investigated by means of a dual mode NC-AFM/STM. Apart from imaging the surface termination by NC-AFM with atomic resolution, point defects in magnesium oxide on Ag(001) and line defects in aluminum oxide on NiAl(110), respectively, were thoroughly studied. The contact potential was determined by Kelvin probe force microscopy (KPFM) and the electronic structure by scanning tunneling spectroscopy (STS). On magnesium oxide, different color centers, i.e., F^0^, F^+^, F^2+^ and divacancies, have different effects on the contact potential. These differences enabled classiﬁcation and unambiguous differentiation by KPFM. True atomic resolution shows the topography at line defects in aluminum oxide. At these domain boundaries, STS and KPFM verify F^2+^-like centers, which have been predicted by density functional theory calculations. Thus, by determining the contact potential and the electronic structure with a spatial resolution in the nanometer range, NC-AFM and STM can be successfully applied on thin oxide ﬁlms beyond imaging the topography of the surface atoms.

## Review

### Introduction

**Figure 1:**
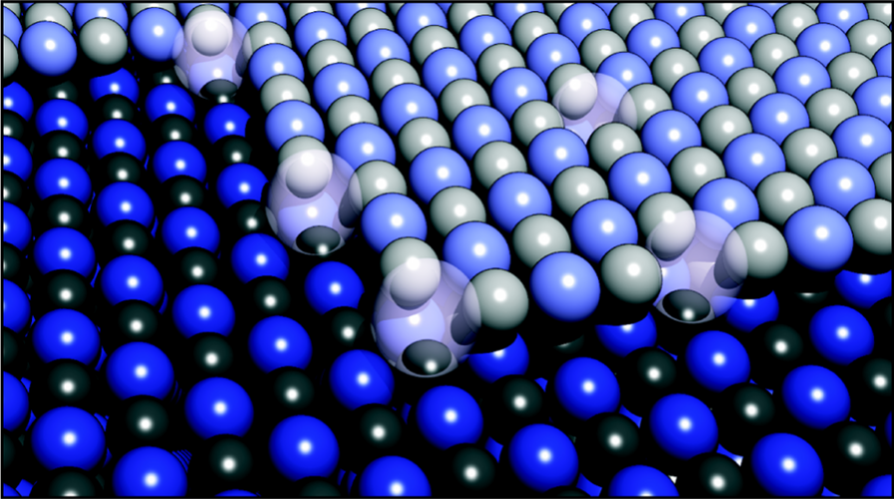
Model of a binary oxide surface. Point defects such as color centers, which are preferably situated at lower coordinated sites, are sketched as bright clouds.

The chemical properties of many crystal surfaces, especially oxides, are signiﬁcantly inﬂuenced by defects in the perfectly ordered structure [1–5]. These defects can be impurities in the surface, interstitials, vacancies or adsorbates. Furthermore, any deviation from the crystalline pattern constitutes such a defect [6]. These defects in the pristine surface may be generated by bombardment with particles, irradiation or contamination with adsorbates. Defects may also be generated during growth. For instance, defects in thin ﬁlms may be caused by a lattice mismatch between ﬁlm and substrate. This may result in a rather frequent and sometimes regular occurrence of the defects. Defect types can be conveniently classiﬁed by the dimensionality of their spatial extension, i.e., as point, line and planar defects. Apart from perturbations of the topography and the stoichiometry, most defects exhibit special electronic structures, which signiﬁcantly differ from the pristine surface. In many cases, it is exactly this deviating electronic structure which produces various special properties of the surface. For example, defects are often preferred adsorption sites and hence are particularly chemically active. Electrically charged defects may enable electron transfer processes, which play an important role in chemical reactions in general and in heterogeneous catalysis in particular. A sketch of a binary oxide surface including several point defects is shown in Figure 1. These point defects could be color centers, where the site of a missing oxygen atom may be empty or occupied by one or more electrons.

In this publication, we review the recent work of our group, where the structure and the topography of defects in oxide surfaces was studied by non-contact atomic force microscopy (NC-AFM) and scanning tunneling microscopy (STM). Furthermore, the contact potential was determined by Kelvin probe force microscopy (KPFM). This technique has a high spatial resolution, thus avoiding averaging over various defects. Here, we conﬁne ourselves to different point defects in magnesium oxide and to line defects in aluminum oxide. Both samples were prepared as thin films on metal supports. As a consequence, STM and scanning tunneling spectroscopy (STS) can be performed and conclusions about the electronic structure of the defects and the pristine ﬁlm can be drawn. This enables a direct comparison with NC-AFM results. The application of NC-AFM and KPFM in combination with STM and STS allows a detailed investigation of the topography as well as of the contact potential and the energetic structure of the defects.

### Experimental setup: dual mode NC-AFM/STM

The employed scanning probe microscope, i.e., a NC-AFM in combination with a STM, was optimized for surface investigation on the atomic scale with spatial resolution of some picometers. Note that NC-AFM is frequently referred to as frequency modulation atomic force microscopy (FM-AFM) or dynamic force microscopy (DFM).

**Figure 2:**
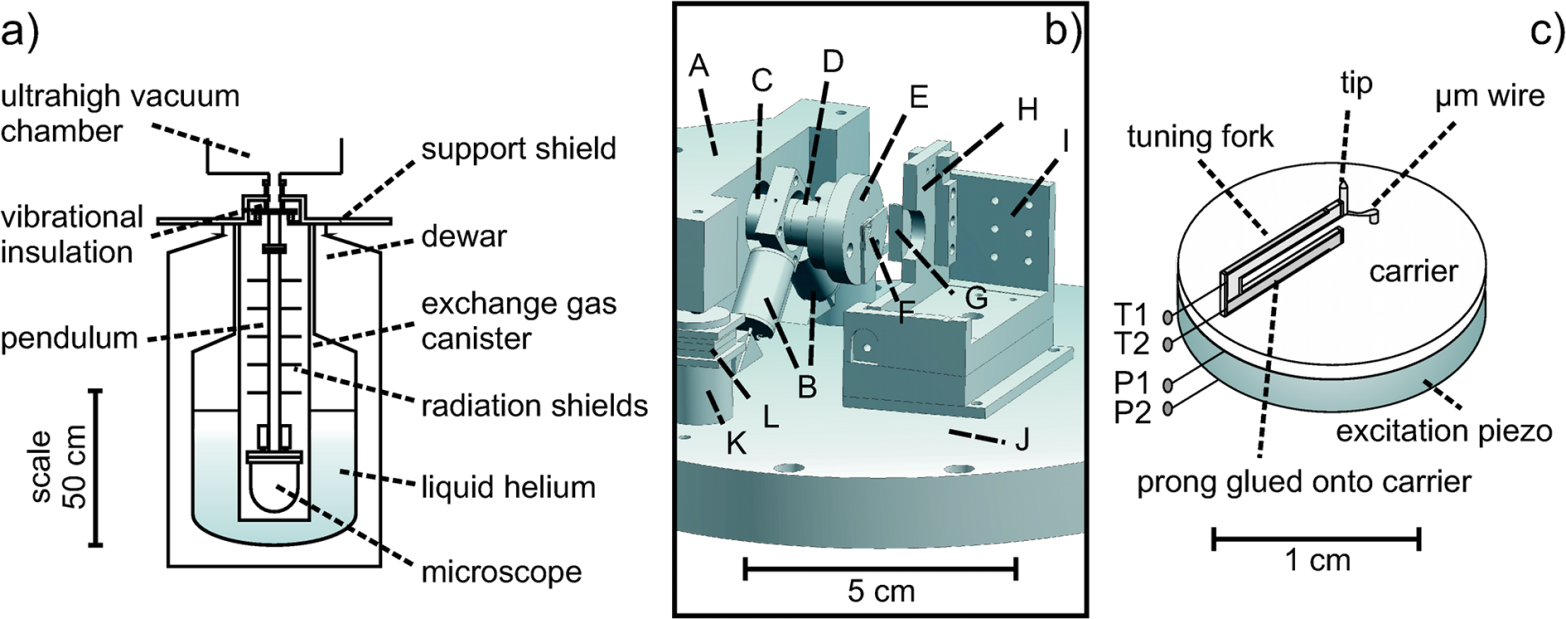
Experimental setup. a) Schematic of an Eigler-style bath cryostat. b) The walker unit is situated on three shear stack piezos for the coarse approach. The *x*, *y* and *z* piezos are used during the scan process. The tuning fork is located opposite the sample (only half of the sample is drawn to keep the view free to the sensor carrier). Schematic of the microscope on its support stage: (A) walker unit, (B) *x*, *y* piezo and (C) *z* piezo of the tripod scanner unit, (D) *z* dither piezo, (E) sensor carrier, (F) tuning fork assembly, (G) sample (not fully drawn), (H) sample holder (not fully drawn), (I) sample stage (not fully drawn), (J) microscope stage, (K) walker support and (L) shear stack piezos. The base plate has a diameter of 10 cm. c) The NC-AFM/STM tuning fork sensor is glued onto the carrier made of MACOR. Contacts P1 and P2 are the contacts of the excitation piezo. The signal from the tuning fork is detected via contact T1 and T2. The µm wire attached to the tip conducts the tunneling current.

For the stability of tip and sample as well as for the reduction of piezo creep, piezo hysteresis, thermal drift and noise level, the setup was operated in ultrahigh vacuum (UHV) at low temperature (5 K). The resulting high stability makes atomic resolution on conductors [7] as well as on insulators [8] possible. In addition to investigations on the surface topography, site speciﬁc spectroscopy measurements can be performed [8]. The whole setup is placed in a sound absorber cabin and is carried on a wooden frame, which, in turn, is based on an active vibrational damping system. The background pressure in the UHV chamber is below 4 × 10^−8^ Pa. The microscope stage is cooled down with a liquid helium bath cryostat (Figure 2a). A so-called exchange gas canister is situated between microscope compartment and helium bath. The exchange gas canister is ﬁlled with helium gas to a pressure of about 1000 Pa. The helium gas establishes thermal coupling between the microscope stage inside the UHV chamber and the liquid helium inside the bath cryostat. In addition, the vibrations caused by the evaporating helium inside the bath cryostat are decoupled from the microscope.

The dual mode NC-AFM/STM sensor (Figure 2c) is situated on a tripod scanner opposite the sample. The scanner, in turn, is mounted onto a coarse approach unit (walker). The microscope stage is shown in Figure 2b. The coarse approach is driven by the shear stack piezos. If the tip-sample distance reaches the range of interatomic forces or the tunneling regime, the walker is switched off and the scan is performed by the *x*, *y* and *z* piezos. An additional excitation piezo orientated along *z* excites the tuning fork at resonance. The tuning fork sensor is presented in Figure 2c. The tuning forks were made of quartz (SiO_2_) and are, therefore, piezo electric devices. Because of their very stable oscillation properties upon electric excitation, they are widely used in watches. Commercial tuning forks have often a resonance frequency of 32768 Hz (=2^15^ Hz). In the employed setup, one prong of the tuning fork is glued onto the carrier. A Pt_0.9_Ir_0.1_ wire, 250 μm in diameter, is attached to the other prong as a tip. The use of a non-conducting glue electrically insulates the tip from the tuning fork and prevents cross talk. Due to the fixed prong and the additional mass of the tip at the other prong, the resonance frequency drops to about 22 kHz.

The tuning fork is driven by the excitation piezo. Due to the piezo electric effect, the signal of the resonance frequency can be detected at the electrodes of the tuning fork. The amplitude of the signal is proportional to the oscillation amplitude of the tuning fork. The signal is so small that a low-temperature amplifier has to be placed nearby to improve the signal-to-noise ratio. In NC-AFM, the shift of the tuning fork resonance frequency Δ*f* is used as a feedback signal to scan with constant Δ*f*. The tip is electrically connected to a wire, 50 μm in diameter (see Figure 2c). Using this electrical contact, a bias voltage can be applied between tip and sample and a tunneling current *I*_t_ can be measured. *I*_t_ serves as a feedback signal when operating in the STM mode at constant current. While operating in one of the modes, NC-AFM or STM, the other channel can always be co-recorded. Great care was taken to ensure that both channels, NC-AFM and STM, were electrically separated from each other in order to prevent cross talk.

**Figure 3:**
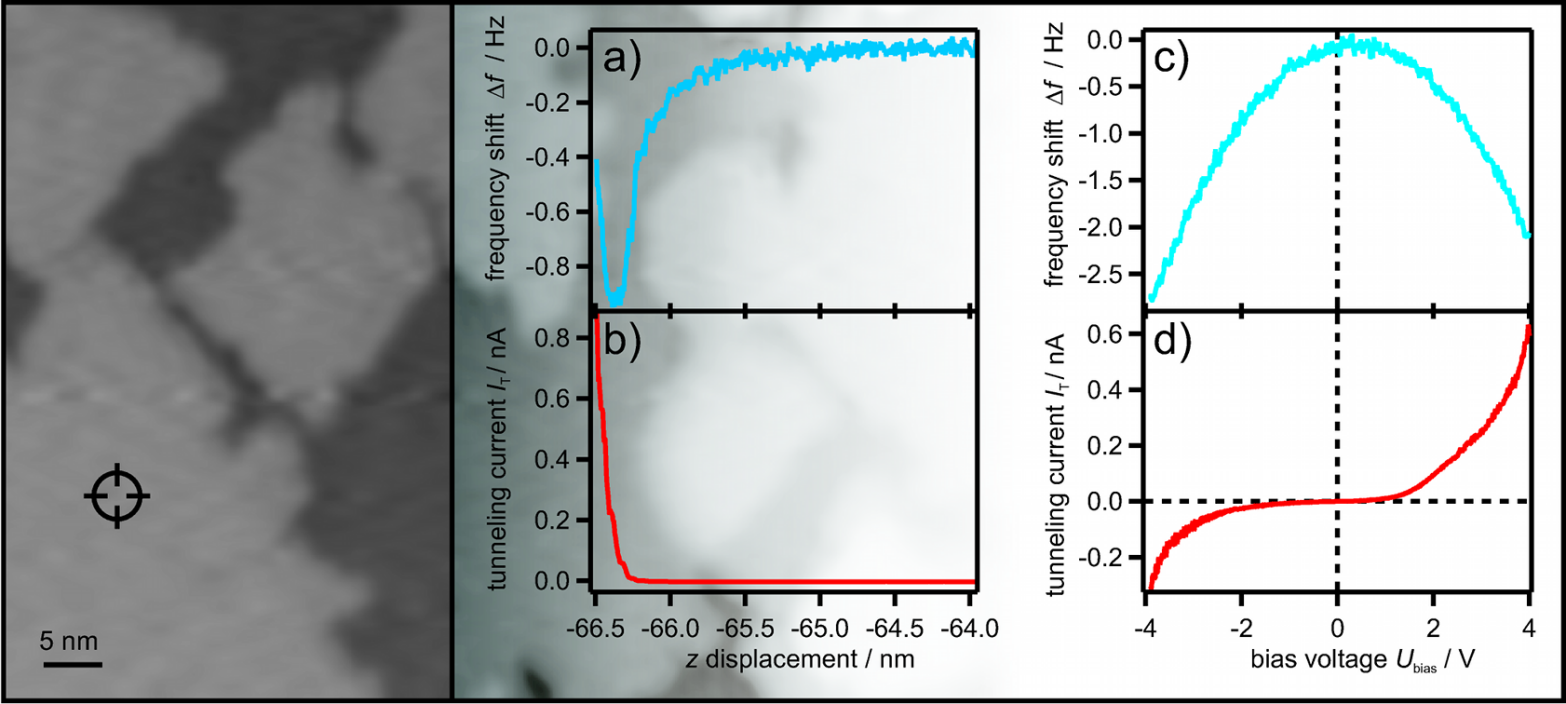
The same tip senses both signals. (a–d) Pairs of simultaneously recorded signal curves from the frequency shift and the current channel: (a,b) signal–distance curves at constant bias voltage, (c,d) signal–bias voltage curves at constant height. On the left-hand side, a STM image of the MgO ﬁlm recorded with a bias voltage of +3.5 V and a tunneling current of 100 pA is shown. The tip position for the spectroscopy is indicated.

The great advantage of this setup is the simultaneous data acquisition of the frequency shift and the tunneling current, making it a powerful tool for high resolution real space analysis at the atomic level and merging the strengths of both techniques. The combination of both techniques enables the detection of contaminants on the tip. For instance, insulating contaminants cause a shift of the minimum of the Δ*f* signal to larger tip-sample distances, whereas *I*_t_ is not influenced. In general, it is interesting to measure both signals as they complement each other and the use of the very same microscopic tip enables direct comparison. Pairs of curves from both channels recorded in a sweep in *z* direction and another one recorded at varying bias voltage are shown in Figure 3.

### Spectroscopic methods: tip-sample forces in NC-AFM

In surface science, forces detectable by NC-AFM in UHV at low temperature have been classified into three main categories [9]. The first category has an electrostatic origin and covers forces between charges, also known as Coulomb forces. These forces arise from the interaction between charges, permanent dipoles and higher order moments. Polarization forces are the second category. These forces cause dipole moments in atoms or molecules, which are induced by electric fields of charges and of permanent or induced dipoles. The third category covers bonding forces, which have a quantum mechanical nature. These forces lead to charge transfer processes as involved in covalent bonding. Furthermore, this category includes the repulsive exchange forces, which are caused by the Pauli exclusion principle. These repulsive forces balance and prevail the attractive forces at very short distances. The classification into these three groups is neither rigid nor exhaustive. For example, van der Waals force, which falls into category two, is a general consequence of the zero-point energy in quantum mechanics [10–11]. Furthermore magnetic forces, friction forces, capillary forces etc. can in principle occur in NC-AFM. These forces are not relevant in this paper, since, e.g., a magnetic tip is necessary to detect magnetic forces, or non conservative forces have to be measured to determine friction forces.

The forces relevant in this work are described below. Coulomb forces are a result of interacting charges and can be stronger than most chemical binding forces [9]. The Coulomb potential *E*_Coulomb_ between two charges *Q*_1_ and *Q*_2_ is given by

[1]
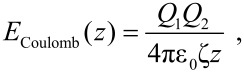


where ε_0_ is the permittivity constant, ζ is the relative permittivity or dielectric constant of the medium and *z* the distance between the charges. The Coulomb force *F*_Coulomb_ is given by

[2]
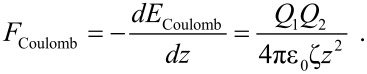


It is well known [12] that for very small amplitudes, the shift of the resonance frequency Δ*f* corresponds to the derivative of the tip-sample forces with respect to *z*. For larger amplitudes, a more general relation can be derived [12], which is not always proportional, however, strictly monotonic. Consequently, the tip-sample forces and potentials can be determined by recording Δ*f* with NC-AFM.

Via detection of electrostatic forces, contact potentials can be determined by NC-AFM in the KPFM mode [13–16], which is named after Lord Kelvin, who measured contact potentials in a similar way [17]. The contact potential (CP) results from the alignment of the Fermi levels of tip and sample having different work functions. The tip-sample geometry can be considered as a capacitor, resulting in the following equation for the electrostatic energy E_el_, which together with the non-electrostatic interaction such as a Lennard-Jones potential adds to the total energy, [18–19]

[3]
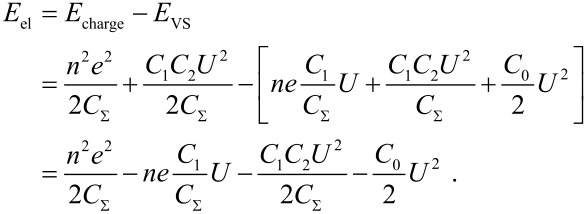


*E*_charge_ is the energy due to electrostatic charging and *E*_VS_ is the work done by the voltage source. Furthermore, *C*_Σ_(*z*) = *C*_1_(*z*) + *C*_2_, with *C*_1_(*z*) is the capacity between the tip and a defect on the surface, *C*_2_ is the capacity between the defect and the substrate and *C*_0_ is the capacity between the tuning fork back electrodes and the surface. The voltage between tip and sample is given by


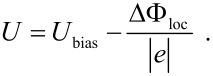


*U*_bias_ is the voltage applied between tip and sample, *e* the elementary charge, ΔΦ_loc_ the local contact potential and *n* represents the number of charges *e*. The derivative of Equation 3 with respect to *z* results in the electrostatic force given by

[4]
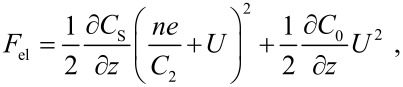


where *C*_S_ is given by a capacitor series *C*_S_ = *C*_1_ · *C*_2_/(*C*_1_ + *C*_2_). The last term in Equation 4 can be neglected when high resolution is considered, since the electrostatic force between the substrate and the tuning fork’s back electrodes integrates a large surface area [18].

In a thin oxide ﬁlm on a metal support, the surface may contain charges. It is reasonable to introduce an effective contact potential ΔΦ_eff_ [20] which considers the shift of the contact potential of the pristine materials due to the charges in the surface, i.e., ΔΦ_eff_ = ΔΦ − *ne*^2^/*C*_2_. This results in

[5]
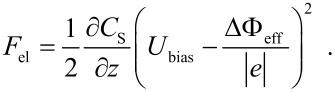


**Figure 4:**
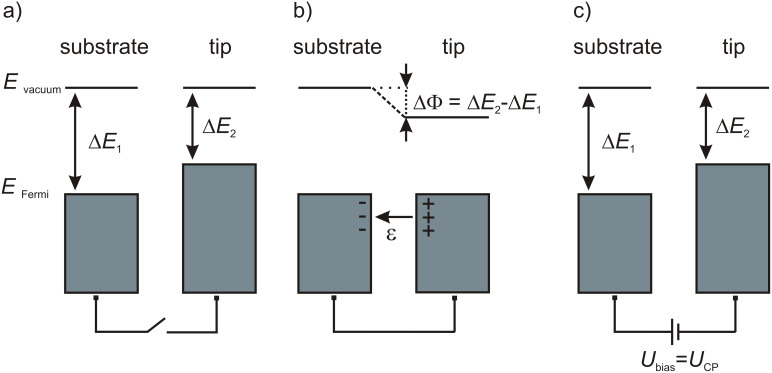
Energetic levels. a) The Fermi levels of tip and sample when they are not electrically connected. b) Tip and sample are electrically connected and the Fermi levels align resulting in an electrostatic field. c) If the sample bias voltage corresponds the contact potential, i.e., *U*_CP_ = ΔΦ/*e*, the electrostatic field is canceled.

Tip and sample are not directly in contact but they are electrically connected via the electronics. The electrical contact leads to an alignment of the Fermi levels of tip and sample. In Figure 4a tip and sample are not electrically connected, thus, the vacuum levels are equal and the Fermi levels do not align. In Figure 4b tip and sample are electrically connected and electrons from the material with the lower work function (here tip) ﬂow to the material with the higher work function. The Fermi levels align and an electrical ﬁeld is built up [21]. The contact potential ΔΦ is then given by the difference in work function of the tip and of the sample surface, which may contain the studied defects. By applying a bias voltage and thus reversing the charge transfer between tip and sample, the effective contact potential can be obtained as the point of minimal force (see Figure 4c). The advantage of KPFM compared with, e.g., photoelectron spectroscopy is the high local resolution down to single point defects or single adsorbates, instead of integrating over a square millimeter range. However, absolute values of the work function cannot be measured directly, only work function differences.

### Point defects

Oxygen vacancies, also known as color centers, are electron trapping point defects and are supposed to be involved in electron transfer processes on the surface. The trapped electrons in the color centers can be transferred to adsorbates such as Au atoms. The defect-free MgO surface is quite inert while a defect rich surface shows a high and complex chemical reactivity [22]. In order to understand possible reaction pathways, a detailed characterization of color centers is highly desirable. Information about their local position and thus coordination, electronic structure, local contact potential and possible adsorbate interaction are of fundamental interest. In the following, color centers on the MgO surface are investigated in detail and classiﬁed by their charge state. From calculations it has been proposed that color centers are directly involved in chemical reactions [23–24], e.g., as adsorption sites due to more attractive defect-adsorbate interactions compared with the pristine MgO surface. It is also experimentally investigated whether color centers are attractive or repulsive in comparison to the surrounding MgO lattice.

#### Sample system: magnesium oxide on Ag(001)

**Figure 5:**
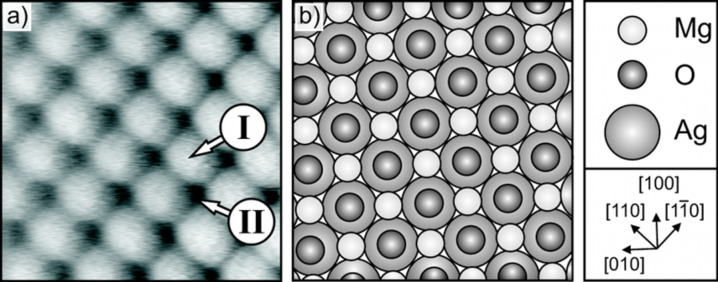
Magnesium oxide surface. a) Atomically resolved image recorded by NC-AFM. The position I and II indicate the two different surface atoms, oxygen and magnesium. The size is 1.5 nm x 1.5 nm and the corrugation approximately 30 pm. Δ*f* = −8.5 Hz, *A*_osc_ = 0.35 nm. b) Schematic growth model of the MgO on Ag(001). The oxygen atoms occupy top sites, while the magnesium atoms occupy hollow sites [8].

An NC-AFM image of a perfect MgO surface is shown in Figure 5. The ﬁlm is two atomic layers thick, however, ﬁlms with a thickness of two to eight layers give very similar images. One type of ion is shown as a protrusion while the other type of ion is depicted as a depression. This is a typical ﬁnding for ionic surfaces imaged by NC-AFM [25–26]. Since the density of electrons on the MgO surface is the highest above the oxygen atoms [27], the maxima in the NC-AFM image are thought to correspond to the positions of the oxygen atoms. Furthermore, electron paramagnetic resonance (EPR) spectra have shown that the preferred adsorption sites for Au atoms are on top of the oxygen ions on the terrace of the MgO surface [26]. Assuming that the forces acting on such metal adatoms are comparable to those on the tip apex, one may conclude that a more attractive interaction occurs between the oxygen sites and the tip. This results in a contrast where oxygen atoms are imaged as protrusions in a constant Δ*f* NC-AFM image.

**Figure 6:**
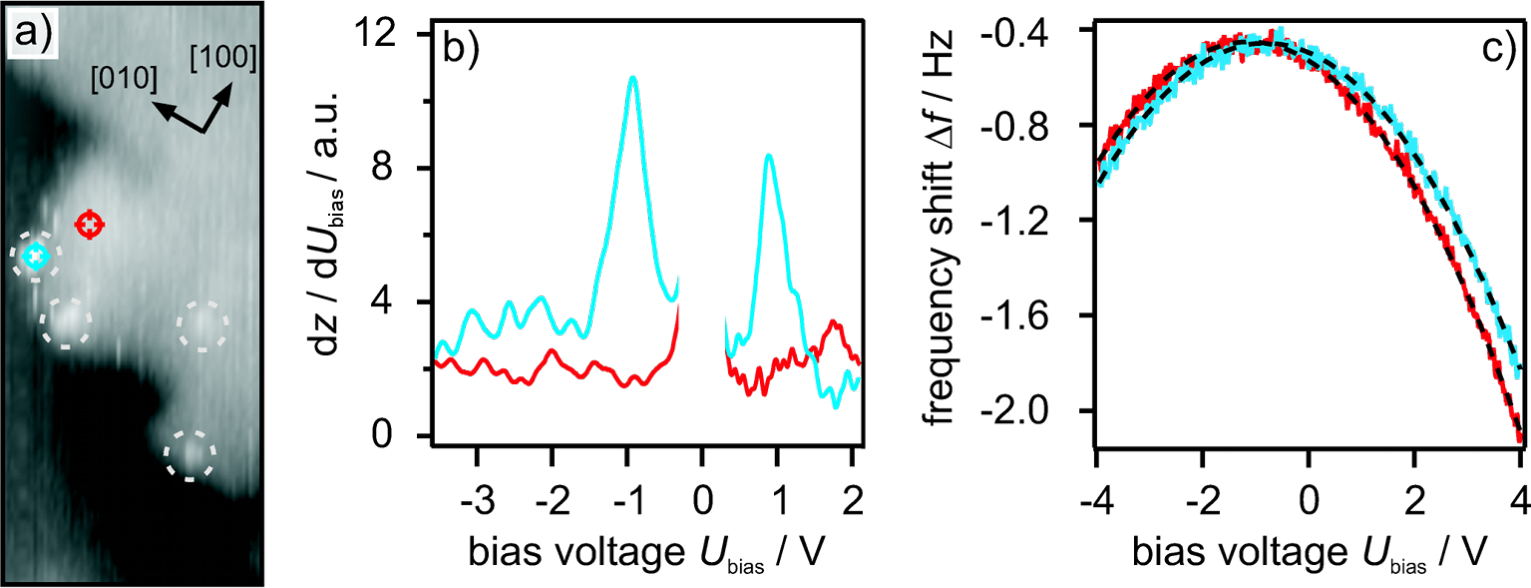
Spectroscopy on point defects. a) NC-AFM image of 21 nm × 9 nm measured at a frequency shift of Δ*f* = −1.6 Hz , an oscillation amplitude of *A*_osc_ = 0.34 nm and *U*_bias_ = −50 mV. Defects are indicated by circles. The position of the spectroscopy in b) and c) is indicated red and blue. b) STS on MgO. There are no states in the MgO-ﬁlm (red), whereas electronic defect states (blue) at approximately +1 V and −1 V exist. c) Frequency shift vs bias voltage spectroscopy shows a quadratic dependence at the MgO-ﬁlm (red) and at the defects (blue). The maxima have different bias voltages.

The preparation conditions of the MgO ﬁlm on Ag(001) follow a route described in [28], where a stoichiometric composition was observed. This procedure has proven its applicability in many successful preparations. The Ag(001) was sputtered with Ar^+^ ions at a current density of 10 µA/cm^2^ and an acceleration voltage of 800 V for 15 min. Afterwards, the Ag(001) was annealed at 690 K for 30 min. The sputtering and annealing cycle was repeated several times. Mg was evaporated from a Knudsen cell in an oxygen atmosphere of 1 × 10^−4^ Pa at a substrate temperature of 560 K and a deposition rate of about 1 ML of MgO/min. A certain amount of MgO can be grown onto the Ag(001) by linear extrapolation of a sub-monolayer coverage to the desired number of monolayers, assuming a constant sticking coefﬁcient. This preparation method is only possible since the reaction kinetics of Ag with oxygen is very slow [29] compared with the reaction between Mg and O. Since the intrinsic defect density of the ﬁlm is very small, color centers, such as F^0^, F^+^ and F^2+^ , have been generated by operating the microscope in the STM mode at high currents *I*_t_ = 6 nA and high voltages *U*_bias_ = 7 V or higher. Clean and well grown MgO areas have been selected to ensure deﬁned conditions. The defects are preferentially located at kinks, corners and step edges (for an illustration see Figure 1). This means defect sites with a lower coordination number are preferred. An NC-AFM image of an MgO step edge with point defects is shown in Figure 6.

### Color centers in magnesium oxide

**Figure 7:**
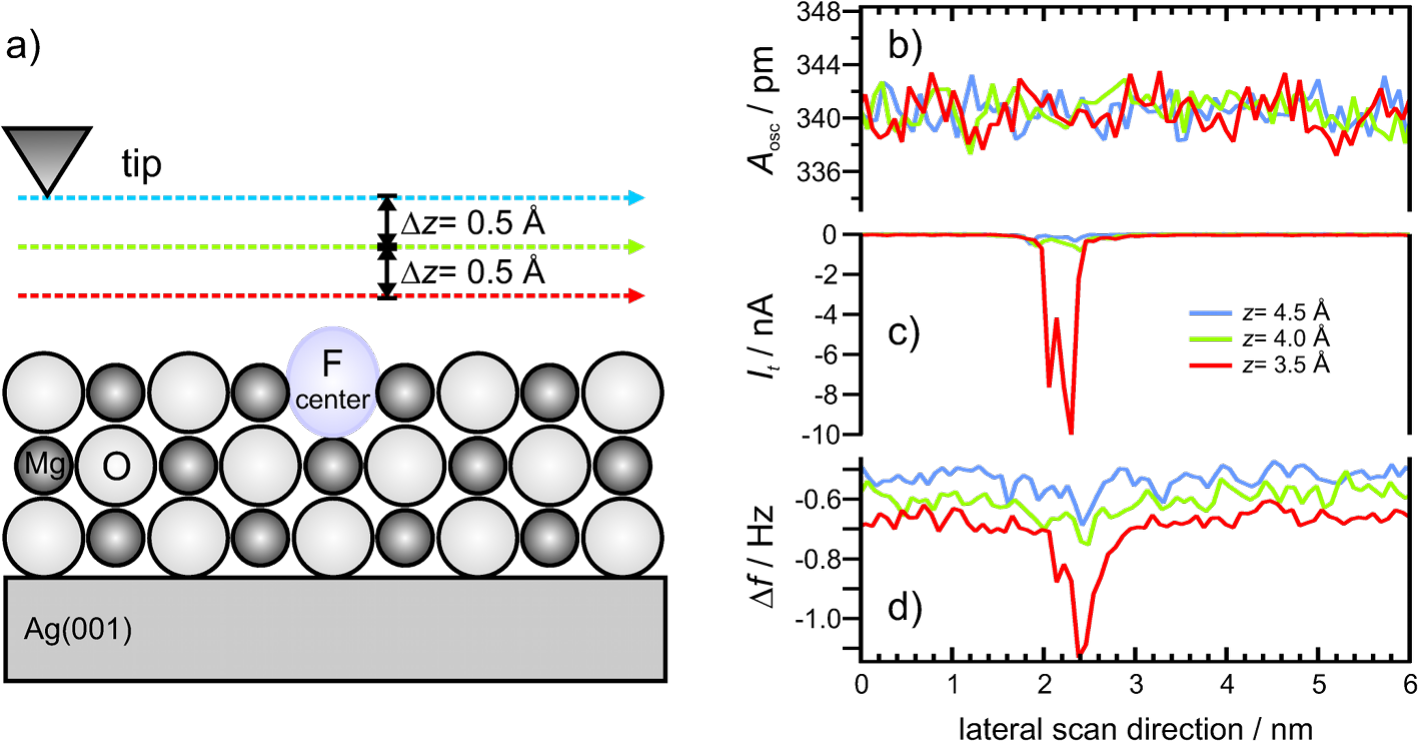
Dependence on tip-sample distance. Constant height line-scans across an F^0^ defect situated at a step edge. The scan direction is along the step edge. The three presented channels have been measured simultaneously. The colors indicate different tip-sample distances. Note that the displacement of 4.5 Å has been chosen arbitrarily, since absolute values are generally unknown in scanning probe microscopy. b) The oscillation amplitude is constant during scan process. This excludes artefacts in frequency shift. c) The tunneling current and d) the frequency shift. Data were obtained at a bias voltage of *U*_bias_ = −50 mV.

The high local resolution of the NC-AFM image shown in Figure 5 serves as the starting point for adsorbate-defect interaction studies. The tip, representing the adsorbate, scans laterally across the defect positions at constant height along the step direction. The simultaneously measured frequency shift Δ*f* and tunneling current *I*_t_ give insight into the local surface potential as well as into the local electronic structure. The corresponding results of such an experiment are shown in Figure 7, where the tip scanned across an F^0^ defect. The three stacked graphs show the simultaneously recorded oscillation amplitude, the frequency shift and the tunneling current. The colored traces indicate constant height scans at different tip-sample separations. At all tip-sample distances the oscillation amplitude can be considered as constant, which is a prerequisite, since the frequency shift scales with the amplitude [12].

Due to the exponential dependence of the tunneling current on the tip-sample distance, *I*_t_ vanishes at the largest separation and the shift of the resonance frequency is a consequence of the long range force background arising from electrostatic and van der Waals forces. The averaged frequency shift at the largest separation is about Δ*f* = −0.52 Hz. By decreasing the tip-sample distance by 0.5 Å, the absolute value of the tunneling current and the frequency shift increase at the position of the defect. The tunneling current increases to *I*_t_ = −0.5 nA and the frequency shift to Δ*f* = −0.75 Hz above the defect. Decreasing the tip-sample separation by another 0.5 Å results in a tunneling current of *I*_t_ = −9.9 nA and a frequency shift of Δ*f* = −1.13 Hz at the defect site. Despite the decrease of 1.0 Å in tip-sample distance, the average tunneling current on the regular MgO terrace remains below *I*_t_ = −0.05 nA. The frequency shift changes by 0.15 Hz with decreasing tip-sample distance. This experiment demonstrates the highly attractive interaction of the tip (or adsorbate) with an F^0^ center.

**Figure 8:**
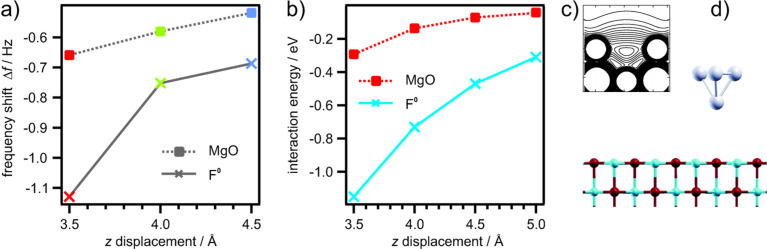
Dependence on tip-sample distance. a) Shift of the resonance frequency of a Pt_0.9_Ir_0.1_ tip on a regular MgO surface (squares) and above an F^0^ defect site (crosses). Experimental data are derived from the constant height measurements shown in Figure 7. The frequency shift is a direct consequence resulting from potential gradients between tip and sample. The integration of the frequency shift is related to the potential energy. b) Interaction energy of a Pt_4_ cluster above the O site of an MgO surface (rectangles) and above an F^0^ defect center (crosses) calculated by DFT. c) The spill over of the electron charge density of an F^0^ center calculated by DFT. d) The Pt_4_ cluster above the MgO surface [35–36].

It has been debated in literature how color centers are imaged by NC-AFM [25,30] since a color center is a hole in the MgO lattice [22]. The observed attraction of F^0^ centers originates from the charge density of the two trapped electrons, which are located in the center of the defect site. Due to Coulomb repulsion, the trapped electrons repel each other and spill out of the defect site into the vacuum [31]. Therefore, a considerably large charge density is situated above the surface. This charge density is supposed to interact with the tip resulting in a strong attraction, as presented in Figure 7. Since the doubly occupied F^0^ state is close to the Fermi level of the MgO/Ag(001) system [32], the charge density is also responsible for the strong peak in the tunneling current signal. Further insights into the interaction of tip and color center are obtained by periodic supercell DFT calculations at the level of the generalized gradient approximation as implemeted in the VASP code, which have been performed in the group of G. Pacchioni [33–35]. The Pt_0.9_Ir_0.1_ tip has been modeled by a tetrahedral Pt_4_ cluster, whose geometry has been relaxed separately. The F^0^ color center has been created by removing an O atom from the top layer of a three layer MgO slab. The structure of the slab with the color center has been relaxed. The tip-surface interaction energy has been computed as a function of tip-sample distance of the apical Pt_4_ cluster with respect to the top layer of the MgO slab (see Figure 8d). During these calculations the separately optimized tip structure was not allowed to relax. However, the relaxation of the MgO surface has been found to be very small for the calculated distances, where no direct contact is established. The outward relaxation of the O anion at 3.5 Å is about 0.12 Å.

**Figure 9:**
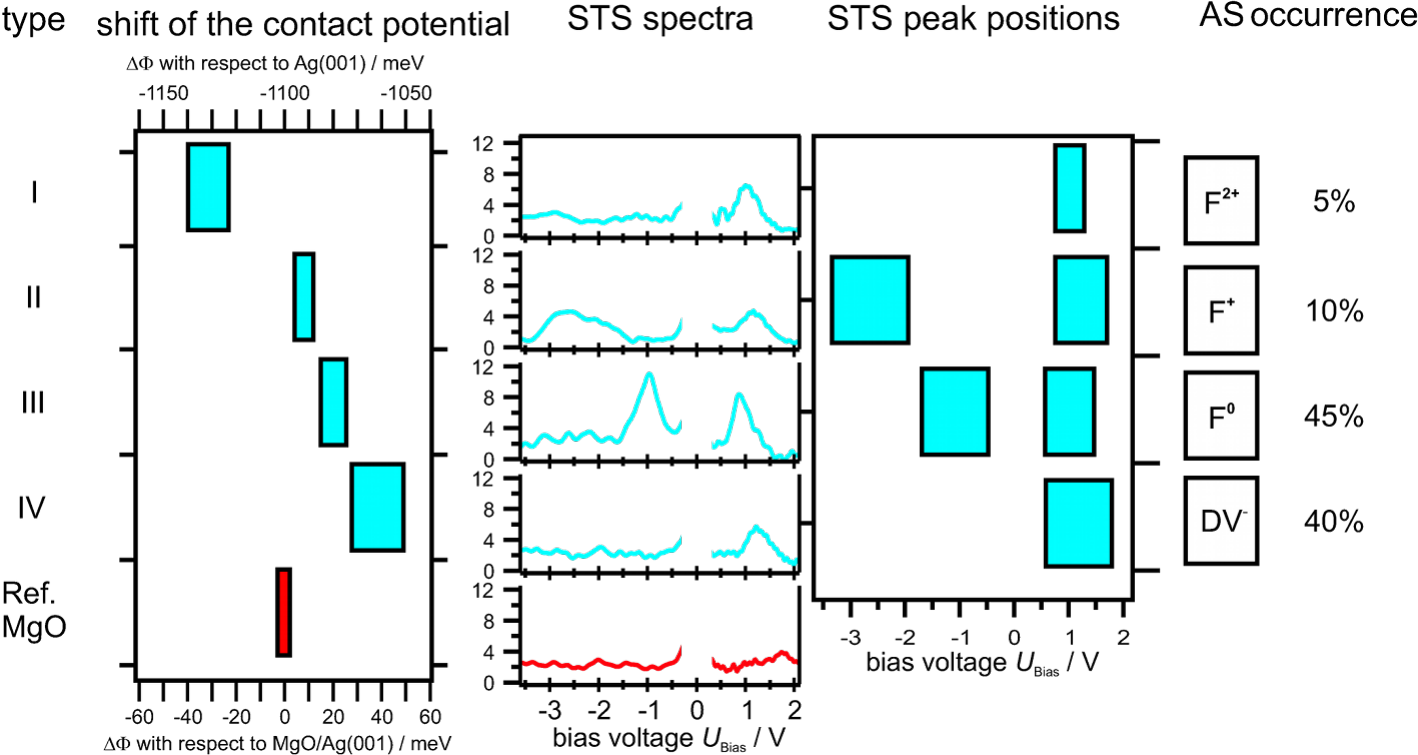
Color centers on MgO. The left labeling assigns numbers to the defect types. The left graph shows the relative shift of the local (effective) contact potential with respect to the MgO surface (bottom abscissa) and with respect to the Ag(001) level (top abscissa). The covered range in the shifts results from measurements with different local resolutions due to different tip structures. The energy level scheme presents the different energy levels of the defect types and their local contact potential shifts. The central graph shows STS spectra of the respective defects. The right graph presents the maxima of the STS data. The covered abscissa range accounts for the statistics of the peak positions. The assignment (AS) of defect types to color centers and negatively charged divacancies (DV^−^) according to theory as well as their relative occurrence are given on the right hand side.

The results of the experimental distance dependent measurements and the corresponding theoretical results are presented in Figure 8. At the defect site, the tip-sample interaction increases signiﬁcantly with decreasing distance. From a structural point of view the positions of the defects are ”holes”, i.e., missing oxygen atoms in the lattice. In the ﬁrst place it is unknown which type of color center, F^0^, F^+^ or F^2+^, is imaged on the MgO surface. To gain further insight into the nature of the color centers we performed high resolution KPFM measurements with single point defect resolution (Figure 6). To acquire Δ*f* vs *U*_bias_ curves on top of a defect, the Δ*f* feedback was switched off. Subsequently the frequency shift vs applied bias voltage was plotted and compared to equivalent reference measurements at the same height close to the defect. The parabolic behavior of the frequency shift curves has been analyzed with Equation 5. The electrostatic force is always attractive. This results in the parabolic dependence of the forces (see Equation 5). The maximum of the parabola depends on the local effective contact potential ΔΦ_eff_. It has been found that the MgO thin ﬁlm shifts the Ag(001) work function and thus the contact potential by about 1.1 eV. This MgO level is set as the reference level and relative shifts are related to it. From measurements of numerous defects four different types were distinguished by their contact potential, which corresponds to the maximum position of the frequency shift vs bias voltage parabola. The results are shown in Figure 9. On the left-hand side of Figure 9 the four types are indicated by numbers and the MgO reference level is given (red bar). The graph on the left-hand side represents the measured contact potential with respect to the reference MgO level (bottom abscissa) and with respect to the Ag(001) level (top abscissa).

For type I defects shifts of −50 to −25 meV below the MgO level were observed. These signiﬁcant shifts can be explained by the presence of positively charged defects with respect to the surrounding area resulting in a decrease of the local contact potential. The charge density distribution is signiﬁcantly reduced at the positions of the defects compared with the surrounding MgO lattice. The presence of charges localized at defect sites induces a contact potential shift of the MgO/Ag(001) in analogy to the Helmholtz equation ΔΦ = 4π*e*µσ [37]. Where µ is the dipole moment induced by the localized charge at the site of the defect and the screening charge in the Ag(001) substrate and σ is the surface concentration. However, the full complexity is not covered by the Helmholtz equation and detailed calculations are still desired. Defect type II shows a contact potential shift of ≈ +9 meV. This shift can be assigned to an F^+^. For an F^+^ the overall charge is positive, but on a very local scale the single electron has a probability above the surface as derived by density functional theory calculations [31]. The charge density spills out of the defect’s site and has therefore a probability above the surface. The spill out of the negative charge changes the local dipole moment such that the local contact potential increases compared with the MgO/Ag(001) reference level. The electron charge is symmetrically distributed along the surface normal with its charge maximum located in the center of the defect. Defect type III results in a shift of about +15 to +20 meV above the MgO level. The shift results from two charges present in a defect site and is thus attributed to an F^0^ color center. An F^0^ is neutral compared to the surrounding MgO lattice, but the two electrons have a large probability density above the surface due to Coulomb repulsion. The charges are as for type II symmetrically distributed and located in the center of the defect, see Figure 8c. Therefore, the charge does not belong to any Mg^2+^ site surrounding the defect. Thus, the oxidation state of the surrounding lattice is not affected by the trapped charges. The spill out of the charges results in a stronger dipole moment compared to defect type II and the measured shift is about twice as large as that for defect type II.

The strongest positive shift on the relative scale is that of type IV. The strong shift indicates that negative charges are involved. Therefore, this shift might result from divacancies (DV) or OH groups trapped at low coordinated Mg^2+^ sites. It is known that OH groups can trap electrons [38]. However, OH groups and other adsorbates can be excluded since all defects occur only after high voltage and high current scanning and are not present on regular terraces and steps. With the above mentioned scan parameters, adsorbates would be removed from the scan area. Furthermore, the defects occur only within the high current scan frame and not outside. Favored candidates are, therefore, divacancies formed at step and corner sites since the formation energy at these sites is the lowest. The stability of divacancies and their electron afﬁnity have been conﬁrmed by DFT calculations [39]. A divacancy is neutral compared with the surrounding MgO, since a complete Mg-O unit is missing. Due to the electron afﬁnity of 0.6–1 eV, electrons can be trapped by the DV from the tunneling junction and the DV becomes negatively charged. The trapped electron of the DV^−^ is strongly localized at the Mg^2+^ site due to the attractive Coulomb interaction. Since the DV^−^ is negatively charged with respect to the surrounding MgO area, the additional dipole moment will increase the work function resulting in the largest positive shift on the relative scale. The covered ranges in the maximum positions originate from different tip structures, however, the reproducibility for two subsequent measurements with the same microscopic tip is within ±2 meV. All defect types analyzed show a characteristic *ﬁngerprint* due to different charge states.

The measurements based on NC-AFM are supported by complementary STS. For all defects the local density of states (LDOS) has been detected. The tunneling spectra measurements have been performed directly after the local contact potential measurements without moving the tip laterally, i.e., STS and KPFM have been performed with the same microscopic tip configuration. To prevent tip changes when carrying out STS at high voltages, the feedback on the tunneling current was switched on and d*z*/d*U*_bias_ was detected. The d*z*/d*U*_bias_ vs *U*_bias_ spectrum at constant tunneling current *I*_t_ is similar to the d*I*_t_/d*U*_bias_ vs *U*_bias_ spectrum at constant height *z*, see [40].

The tunneling spectra measured on the defects are compared with MgO spectra on the terrace next to the defect. The MgO reference spectra show no peaks within the voltage regime due to the band gap (compare red lines in Figure 9). The spectra taken on the F^2+^ only show peaks in the unoccupied regime at voltages of ≈ +1 V above the Fermi level (see Figure 9). The F^+^ centers have both occupied and unoccupied electronic states within the band gap. The electronic states are located within the band gap of MgO. The occupied states are quite broadly distributed from −3.5 V to −2.0 V below the Fermi level, depending on the defect location on the ﬁlm [32]. The empty states are at ≈ +1 V above the Fermi level. Considering the F^0^ color center, the doubly occupied state is higher in energy, approximately −1 V below the Fermi level, while the position of the unoccupied state is similar to F^+^ centers.

The negatively charged divacancies only show a clear feature in the empty states at about +1 V. The corresponding occupied shallow state is expected to be very close to the Fermi level, i.e., in a region where the experiment cannot clearly detect states. However, F^0^ and DV^−^ are equally frequent and represent ≈85% of the total defects. F^+^ color centers are much less frequent and represent ≈10% and F^2+^ centers about 5%. These ﬁndings are in good agreement with the high formation energies of F^2+^ centers. By comparing the STS peak positions in Figure 9, it becomes obvious that F^2+^ and DV^−^ defects are hardly distinguishable by their electronic structure but show a signiﬁcant difference in the local contact potential due to the effect of a locally trapped charge on the surface dipole. This demonstrates the great beneﬁt of NC-AFM and KPFM in combination with STM and STS.

### Line defects

Apart from point defects more complex structures like line defects are found on oxide surfaces. Line defects can be caused by step edges or grain boundaries that penetrate the surface. In thin oxide ﬁlms line defects are often generated by domain boundaries. The structure at these line defects usually differs signiﬁcantly from the defect-free domains. This is often associated with a change of electronic properties, which may signiﬁcantly inﬂuence the surface chemistry.

#### Sample system: aluminum oxide on NiAl(110)

Thin ﬁlm aluminum oxide on NiAl(110) is composed of two oxygen and two aluminum layers limiting the ﬁlm thickness to 0.5 nm [41]. It is prepared in a reliable and simple two step oxidation procedure. After dosing 5 × 10^−4^ Pa oxygen at 550 K for 10 minutes, the sample is heated to 1050 K in vacuum to crystallize the oxide ﬁlm. This process may be repeated to close open metal patches in the ﬁlm. The preparation is explained in detail in [42]. The ﬁlm grows in two reﬂection domains, A and B. The long edges of the parallelogram shaped unit cells (1.055 nm × 1.788 nm, α = 88.7^◦^) are rotated by ±24^◦^ with respect to NiAl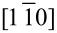.

#### Antiphase domain boundaries in aluminum oxide

The most common structural defects in the thin ﬁlm aluminum oxide on NiAl(110), besides substrate induced step edges, are reﬂection domain boundaries (from domain A to B or vice versa) and antiphase domain boundaries (abbrev. APDBs; A-A or B-B). The latter are translation domain boundaries originating from strain relief and introduced into already existing oxide patches. For this ﬁlm system their denotation as APDBs is common usage due to historical reasons and to distinguish them from boundaries between nucleation related translation domains. While the reﬂection domain boundaries occur less frequent, APDBs occur regularly, approximately every 8–10 nm to release stress in the aluminum oxide ﬁlm that accumulates due to a small lattice mismatch with the NiAl(110) surface along the 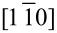 direction.

Different types of APDBs exist, the most common types are straight (type I) and zigzagged (type II) APDBs [43–44]. At straight APDBs the surface unit cell is extended parallel to the long edge of the aluminum oxide unit cell. At zigzagged APDBs both directions of the oxide unit cell are extended. For the sake of simplicity, we focus on straight APDBs in this section. A more comprehensive NC-AFM study of the ADPBs and other line defects on aluminum oxide in NiAl(110) can be found in [43,45–46]. By DFT calculations [47], the stoichiometry of the ﬁlm with a straight APDB was determined to be (NiAl)^2−^_substrate_ (Al_19_O_28_Al_28_O_32_)^2+^. An oxygen deﬁciency with unoccupied electronic states in the aluminum oxide band gap was proposed.

**Figure 10:**
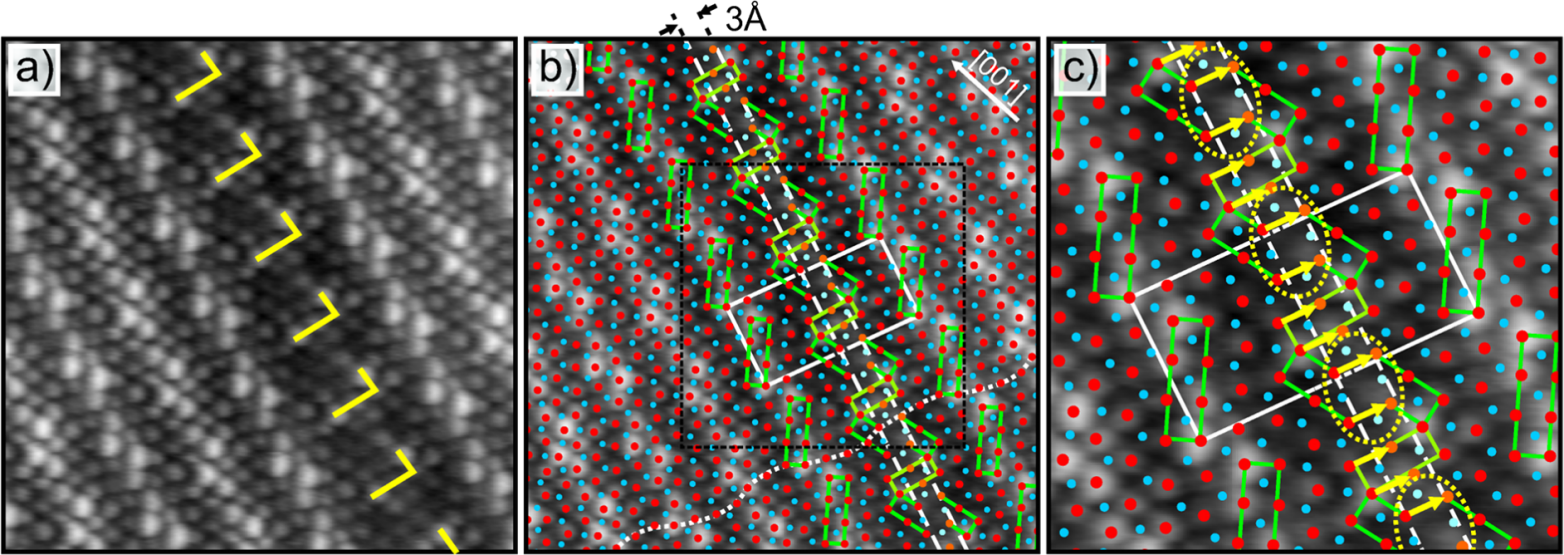
Atomic resolution NC-AFM image of a straight antiphase domain boundary (type I) in the aluminum oxide on NiAl(110). The scan area is 6.4 nm × 6.4 nm in (a) and (b). b) An adjusted model [47] has been superimposed. The unit cell is extended by 3 Å along the long edge of the unit cell. Inserted sites are given in lighter colors. Dashed lines indicate the extension. The dotted line highlights wave-like oxygen rows along the unit cell. c) shows an enlarged section of the image for better visibility (3.5 nm × 3.5 nm). Yellow arrows denote the direction and length (3 Å) of the Burgers vector. Yellow loops indicate spacious arrangements of oxygen sites that are different from all domain sites. Δ*f* = −2.75 Hz, *A*_osc_ = 3.8 Å, *U*_bias_ = −220 mV.

An atomically resolved NC-AFM image of a straight APDB (type B I) is shown in Figure 10. Clearly visible, the boundary is marked by a fairly wide linear depression. The adjusted model for the lateral positions at the APDB [47] is superimposed in Figure 10b and found to be in perfect agreement. From this we see that NC-AFM images the surface oxygen sites of the ﬁlm with high accuracy. The model is based on a unit cell that has been split in the middle according to STM images. Important structural elements of the oxygen sub-lattice are highlighted as well as the extended unit cell and two equivalent lines between which the inserted new sites are visible. Inserted sites are marked in a slightly different color to distinguish them from the usual sites in the oxide unit cell: orange and light blue as compared to red and blue. In Figure 10c an enlarged section of the elongated unit cell at the APDB is given. In the middle of the APDB a broken block of 8 O atoms appears, which is of the type that is almost aligned with the NiAl[001] direction. A particularly spacious arrangement of oxygen atoms in the shape of a quadrangle (yellow dotted loops) is formed at this block at the boundary. This is in agreement with DFT calculations [47], which assign an electronic defect state to this structure. Another deviation from the usual oxide unit cell is a rectangle of six oxygen sites which is derived from the bridging square groups indicated in light green in Figure 10c. These characteristic protrusions in the topography of the boundary form the shape of the letter ’L’ as indicated by the yellow angle in Figure 10a.

**Figure 11:**
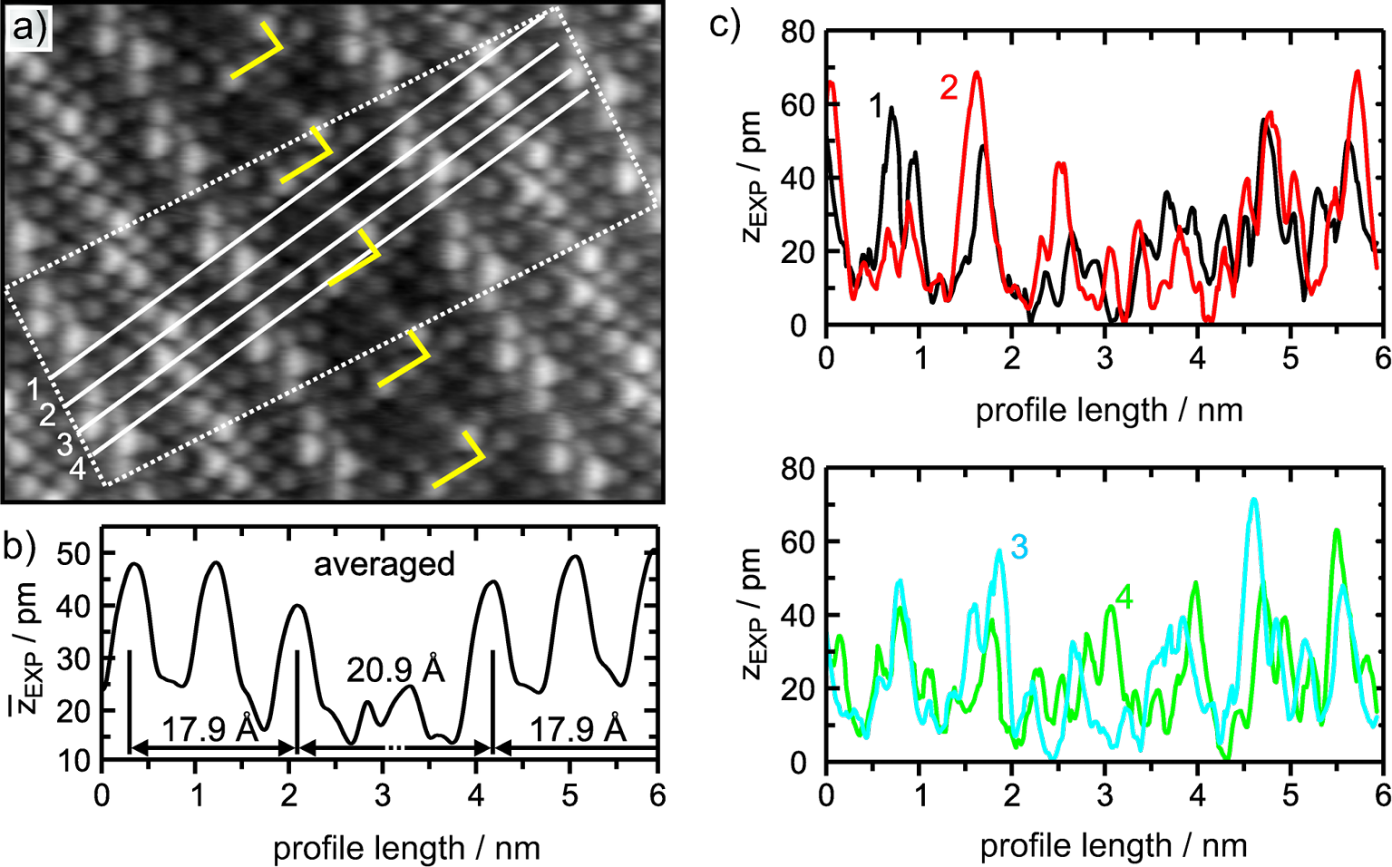
Height proﬁles. a) Cutout from Figure 10. White lines indicate positions where line proﬁles have been taken across the type I boundary. b) Averaged line proﬁle taken within the rectangle (two unit cells in width) in the frame above. This emphasizes that such boundaries are reproduced as depressions within NC-AFM images. Averaging was performed over 167 line proﬁles. c) Single proﬁles along chains of O atoms across the APDB marked by white lines in the rectangle in (a). Such atom rows show heights different from average terrace height. Only the proﬁle labeled 4 shows nearly no decrease in height over the APDB.

The direction and the length of the lattice discrepancy generated by a dislocation in a crystal is given by the Burgers vector. At straight APDBs this vector measures 3 Å in length and is parallel to the long edge of the oxide unit cell as indicated by yellow arrows. At the same time the Burgers vector is also parallel to the overall direction of the wave-like rows of atoms within the surface aluminum and oxygen sub-lattices (dotted line in Figure 10b). Considering the topographic quality of the contrast, the domain boundary can ﬁnally be determined to be a depression. This is summarized in Figure 11. An averaged line proﬁle across the APDB I covering the width of 2 unit cells, as indicated by the rectangle in Figure 11a, is shown in Figure 11b. In Figure 11c individual line proﬁles across oxygen rows are shown. These proﬁles have been taken along the white lines 1, 2, 3, 4 in the image in (a). At the linear oxygen rows there exist sites with a mean height that is 10 pm lower than the average height of corresponding terrace sites (see Figure 11b).

**Figure 12:**
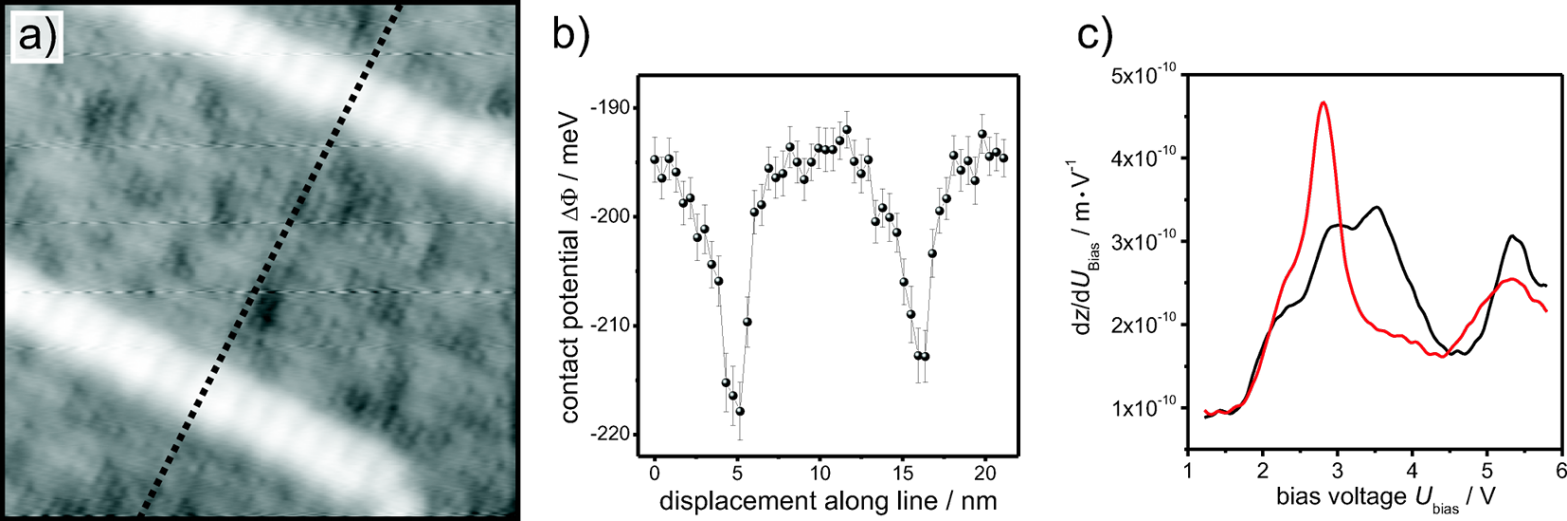
Spectroscopy on aluminum oxide. a) STM image of a thin ﬁlm of aluminum oxide on NiAl(110), 18 nm × 18 nm. Two straight APDBs (bright) separating three A domains (dark) are visible. *U*_bias_ = +3 V and *I*_t_ = 100 pA. b) Effective contact potential, which was determined at the line shown in (a), decreases at the APDB by approximately 20 meV. The tip height was constant during the measurement and corresponds to a frequency shift of −1 Hz at −150 mV. The contact potentials were determined by ﬁtting the frequency shift vs bias voltage curves with a quadratic term (see Equation 5). The error bars represent the accuracies by which the maxima were determined. c) STS curves taken at the domain (black) and the APDB (red).

Knowing the surface structure with highest accuracy, it is still very desirable to determine aspects of electronic structures to gain further insight. In Figure 12, the effective contact potential is plotted for positions along a line across three domains and two straight APDBs. The recorded contact potential at the APDB is approximately 20 meV smaller than at the regular domain, which was veriﬁed at many different sites [45]. This means the work function at the APDBs is reduced compared to that of the domain. Comparison of STS curves on domain and APDB shows signiﬁcant differences in the electronic structures. At the domain boundary a pronounced unoccupied defect state appears at bias voltages between 2–3 V.

The real variation of contact potential may be even larger, since the recorded signal is a convolution of the actual contact potential difference with the tip geometry [48]. The depressions in the contact potential at the APDBs have approximately a full width at half minimum of 3 nm (see Figure 12b). The lateral extension of the APDBs is approximately 1.5 nm and the oxide unit cell is expanded by an additional row of oxygen atoms by 0.3 nm at that position [41]. Assuming the change of the contact potential to be approximately located in this range (between 0.3 and 1.5 nm), the recorded contact potential is broadened by a factor of 2 to 10 due to the convolution with the tip geometry. This means on the other hand, the contact potential difference is actually 2 to 10 times larger than recorded. Furthermore, we conclude that the tip is inﬂuenced by these defects over a distance of approximately 2–3 nm, which might be a reasonable estimation of the tip size. This gives also the lateral resolution of the measurements. As shown in [36] and [49], the determined contact potential difference depends also on the tip-sample distance. In general, a smaller distance increases the size of the interaction and decreases the integration area, this means the determined difference of the contact potential increases. If the distance is too small, the probability that the tip restructures increases. Therefore, the tip-sample distance was set to a moderate value which corresponds to roughly 0.5–0.75 of the maximum absolute frequency shift.

Thin ﬁlm aluminum oxide reduces the work function on NiAl(110) by approximately 0.5 eV from 4.8 eV for a pure NiAl(110) surface to 4.3 eV for the aluminum oxide ﬁlm [50]. A further reduction of the work function at the APDBs may explain the higher reactivity at these linear defects. In [51], it has been shown that APDBs are preferred adsorption sites for different atoms and metal clusters. Furthermore, a particular chemical activity at the APDBs has been experimentally veriﬁed. For instance, nitric oxide decomposition on thin ﬁlm aluminum oxide preferentially takes place at the APDBs [52].

It has been predicted by means of DFT calculations [47] that unoccupied defect states in the APDBs of the aluminum oxide ﬁlm are associated with F^2+^-like centers. In our NC-AFM measurements, we have recorded a shift of the local work function of approximately −20 meV at the APDB. This is in great agreement with the shift recorded at F^2+^ centers on MgO/Ag(001) (see Figure 9). As it has been shown above, a small shift of the contact potential difference is caused by the fact that the recorded contact potential depends on the tip-sample distance. The recorded change of the work function is in agreement with the DFT calculations, where a shift of the valence and the conduction band with a local band bending at the APDB were predicted [47]. Thus, in the APDB F^2+^-like centers, which have been predicted by DFT calculations, are now experimentally veriﬁed by NC-AFM.

## Conclusion

Defects on surfaces of thin oxide ﬁlms were studied by means of low temperature NC-AFM combined with STM in UHV. In addition to imaging the topography of the surface termination, STS and KPFM were employed for a deeper insight into the nature of the defects. The spectroscopy was performed with a very high spatial resolution in the order of 1 nm. For magnesium oxide on Ag(001), different point defects, which are the most frequently discussed ones in literature, were studied. Using contact potential measurements by KPFM in comparison to STS spectra and DFT calculations, the point defects on an MgO surface could be unambiguously identified for the ﬁrst time. The point defects were distinguished as DV^−^, F^0^, F^+^ and F^2+^ color centers. In addition, the electronic signature was measured and electronic defect states were determined within the band gap of the MgO surface. These color centers inﬂuence the surface chemistry by signiﬁcantly increasing the reactivity of the almost inert surface of defect-free MgO. The NC-AFM investigation on aluminum oxide on NiAl(110) unveils the surface structure of the domain and at the APDBs with atomic resolution. Apart from the determined topography, F^2+^-like centers, which have been predicted by DFT calculations, were experimentally veriﬁed for the APDBs. These studies show that NC-AFM in combination with STM can be successfully used beyond imaging the topography of the surface termination. The employed high resolution spectroscopy signiﬁcantly improves our understanding of the surface chemistry of thin oxide ﬁlms.
